# Adaptation of the MapMan ontology to biotic stress responses: application in solanaceous species

**DOI:** 10.1186/1746-4811-3-10

**Published:** 2007-09-04

**Authors:** Ana Rotter, Björn Usadel, Špela Baebler, Mark Stitt, Kristina Gruden

**Affiliations:** 1 National Institute of Biology, Večna pot 111, 1000 Ljubljana, Slovenia; 2 Max Planck Institute of Molecular Plant Physiology Am Mühlenberg 1, 14476 Golm, Germany; 3 Jožef Stefan Institute, Jamova 39, 1000 Ljubljana, Slovenia

## Abstract

**Background:**

The results of transcriptome microarray analysis are usually presented as a list of differentially expressed genes. As these lists can be long, it is hard to interpret the desired experimental treatment effect on the physiology of analysed tissue, e.g. via selected metabolic or other pathways. For some organisms, gene ontologies and data visualization software have been implemented to overcome this problem, whereas for others, software adaptation is yet to be done.

**Results:**

We present the classification of tentative potato contigs from the potato gene index (StGI) available from Dana-Farber Cancer Institute (DFCI) into the MapMan ontology to enable the application of the MapMan family of tools to potato microarrays. Special attention has been focused on mapping genes that could not be annotated based on similarity to Arabidopsis genes alone, thus possibly representing genes unique for potato. 97 such genes were classified into functional BINs (i.e. functional classes) after manual annotation. A new pathway, focusing on biotic stress responses, has been added and can be used for all other organisms for which mappings have been done. The BIN representation on the potato 10 k cDNA microarray, in comparison with all putative potato gene sequences, has been tested. The functionality of the prepared potato mapping was validated with experimental data on plant response to viral infection. In total 43,408 unigenes were mapped into 35 corresponding BINs.

**Conclusion:**

The potato mappings can be used to visualize up-to-date, publicly available, expressed sequence tags (ESTs) and other sequences from GenBank, in combination with metabolic pathways. Further expert work on potato annotations will be needed with the ongoing EST and genome sequencing of potato. The current MapMan application for potato is directly applicable for analysis of data obtained on potato 10 k cDNA microarray by TIGR (The Institute for Genomic Research) but can also be used by researchers working on other potato gene sets. The potato mapping file and the stress mapping diagram are available from the MapMan website [1].

## Background

The output of microarray statistical data analysis is often in the form of a list (or lists) of differentially expressed genes. Depending on the null hypothesis that is being tested and the experimental treatments that have been carried out, these lists can vary in length but often enough these are too long for a rigorous manual inspection. This poses a problem of complexity of interpretation. Lists can be condensed by organising them according to their known or suspected function, but this requires gene ontologies, which cannot be automatically extended from one organism to another, especially when they are not closely related. In addition, transcriptome data analysis is being combined with proteome (or metabolome) data analysis which only adds to the complexity of interpretation. Development of new, more reliable methods of data analysis and visualization will enable easier interpretation of results and thus a greater contribution to explaining the biological problem. Various visualization tools that help the data analysts and the biologists are available today, from GenMapp, Pathway Processor and GeneXpress (reviewed in [2]) to KaPPA-View [3] and VANTED [4]. Using them, it is possible to find trends that would be less apparent from using only lists of genes [5]. While they are suitable for some organisms, their usefulness for plant organisms can be restricted because they have often been developed for microbial or animal systems and thus have categories that are irrelevant for plant systems and lack plant-specific pathways and processes [5,6].

### MapMan organization

A plant-specific visualization tool, MapMan, has been developed to overcome this problem. Initially it was developed for genes from *Arabidopsis thaliana *that are present on the Affymetrix 22 K array [6]. Its main purpose is to organize and display experimental data sets or results onto diagrams of the users' choice [5,6]. It consists of two modules, (*i*) Scavenger module and (*ii*) ImageAnnotator.

The Scavenger module is a gene ontology, in which genes are assigned based on their annotation into largely non-redundant and hierarchically organised BINs. Each BIN consists of items of similar biological function and can be further split into subBINs, corresponding to submodes of the biological function [7]. The original BIN assignments for *A. thaliana *were based on publicly available gene annotations from TIGR (The Institute for Genomic Research) using a process which involved alternation between automatic recruitment and manual correction [6]. The resulting BINs are shown in Table 1; these are broken down by current versions into > 1200 subBINs.

**Table 1:** MapMan BINs. Description of MapMan BINs. The percentage of all clones in StGI present in a specific BIN is shown. The number of clones present on the microarray is also denoted. BINs written in bold are completely covered on the potato 10 k microarray, meaning that each subBIN has at least one representative on the potato 10 k microarray. Note: percentages may not sum to 100 due to rounding errors.

**BIN code**	**BIN name**	**% of all StGI clones**	**number on microarray**
0	control genes	0.2	96
1	photosynthesis	0.8	197
2	major carbohydrates	0.5	90
3	minor carbohydrates	0.4	79
**4**	**glycolysis**	0.3	63
**5**	**fermentation**	0.06	11
6	gluconeogenesis/glyoxylate cycle	0.02	7
**7**	**oxidative pentose phosphate pathway**	0.1	25
8	TCA cycle/organic acid transformations	0.3	57
9	mitochondrial electron transport/ATP synthesis	0.4	82
10	cell wall	1	200
11	lipid metabolism	1	241
12	nitrogen metabolism	0.08	17
13	amino acid metabolism	1	296
**14**	**sulphur assimilation**	0.05	13
**15**	**metal handling**	2	44
16	secondary metabolism	1	266
17	hormone metabolism	2	274
**18**	**cofactor and vitamin synthesis**	0.1	21
19	tetrapyrrole synthesis	0.2	45
**20**	**stress**	3	489
21	redox	0.6	133
22	polyamine metabolism	0.08	25
23	nucleotide metabolism	0.5	113
**24**	**biodegradation of xenobiotics**	0.06	11
**25**	**C1 metabolism**	0.1	20
**26**	**miscellaneous enzyme families**	4	605
27	RNA	7	1174
28	DNA	1	194
29	protein	9	1722
30	signalling	3	521
**31**	**cell**	2	300
**33**	**development**	1	234
34	transport	2	375
35	not assigned	55	6143

The ImageAnnotator module uses the classifications from the Scavenger module in the form of mapping files in order to display data on various diagrams of the user's choice [5]. A mapping file for an organism includes, but is not limited to, these categories: BIN code; BIN name; identifier; description. The identifier includes gene names or clone names with their descriptions, i.e. the names which link the mapping file with the experiment file.

The ImageAnnotator also uses diagrams for data display. They are obtained with the MapMan software [1] or can be self-made and then included in MapMan as described in [5].

### Potato annotation

The potato genome has yet to be fully sequenced. Thus, at present only an approximation of the potato transcriptome is available in the form of the potato gene index that contains expressed sequence tags (ESTs) from diverse tissues. The potato gene index (StGI [8]) clusters ESTs into tentative contigs (TCs) after removing low quality ESTs [9]. The information in StGI is constantly being updated, with 13 releases since July 2000. Not all gene functions within gene indices are well characterized. The StGI database contains clone sequence data (in GenBank) and the corresponding tentative consensus sequence (TC) of the clones that theoretically represent one gene.

The TIGR potato 10 k array is the only microarray platform that is currently publicly available for researchers studying potato. Together with several control clones, the microarray contains 15,264 clones. Each cDNA clone is spotted in two spatially separated replicates thus summing up to 30,528 spots on each microarray. For a general purpose microarray design it is expected that clones from every BIN should be present on the microarray.

We found MapMan implementation for potato beneficial as it will facilitate biological interpretation, support an ontology-based statistical data analysis (see e.g. [10]) and provide users a global overview of the results. We have also added a new mapping of plant's response to biotic stress in order to facilitate the studies of biotic interactions.

## Results and Discussion

Given the usability of potato microarrays in various potato experimental systems, [11-13], we have implemented MapMan to visualize potato transcriptomic data. The sequences and the annotations of the potato gene index (Solanum tuberosum gene index, StGI) version 10 were BLASTed against Arabidopsis proteins (release TAIR 6). In this way, every potato clone was assigned up to ten best matching Arabidopsis proteins.

The Arabidopsis proteome was chosen for comparison because (*i*) the original MapMan Scavenger module was constructed for Arabidopsis, (*ii*) the Arabidopsis genome has been fully sequenced and (*iii*) potato is phylogenetically most similar to Arabidopsis among plant species with known genomes. BLASTing results were put into a file which was modified in order to contain the potato unigene name and its annotation from StGI, the protein domain description from StGI, the best matching Arabidopsis gene names and the corresponding E value, and information on the presence of the clone on the TIGR potato 10 k microarray, and the BIN assignment from Arabidopsis in order to match the original BIN assignment for every gene name. Potato clone annotations were checked manually with the matching Arabidopsis entry; if it differed and the E value was reasonably low (threshold 10^-15^), the BIN assignment was left as for Arabidopsis. When E values were higher than the chosen threshold (between 10^-10 ^and 10^-15^), clones were assigned manually to corresponding BINs, on the basis of sequence and literature searches.

Some of the problems encountered in converting MapMan for a potato clone set were due to species-specific differences in the metabolism. Further, potato is a model organism for plant-pathogen interactions and for physiological processes like tuberization, dormancy and sprouting [14]. Therefore expert input is needed for more detailed BIN structuring and for preparing schemes for such specific processes.

The final mapping file had 43,408 entries that represent the 38,239 different sequences in the potato gene index; of these 15,817 (around 36%) are present on the TIGR potato 10 k microarray. The percentage of clones in a BIN compared to all StGI clones, as well as the numbers of clones from a BIN that are present on the 10 k microarray, are shown in Table 1.

### Classifying biotic stress responses in potato

**Figure 1:**
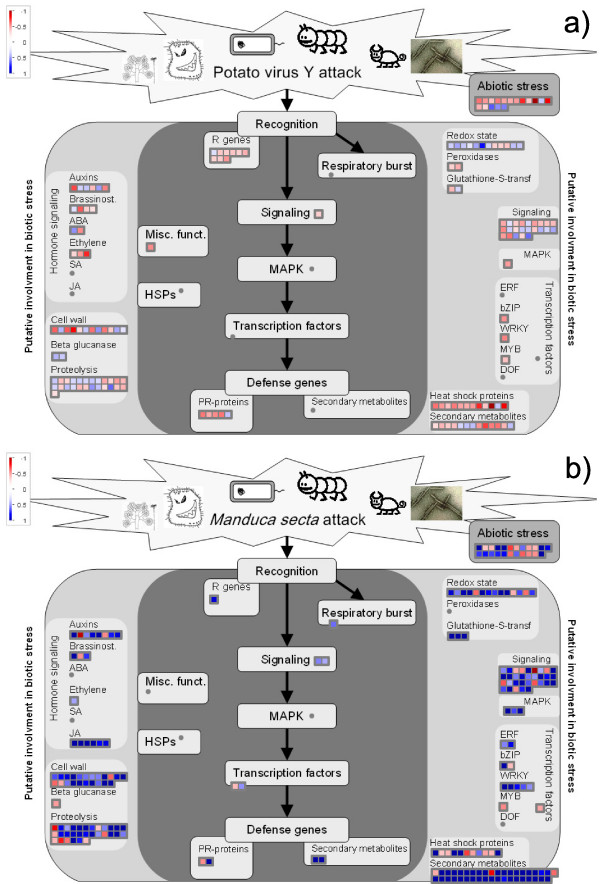
**Changes in expression during responses of plant samples to pathogens**. The plant's reaction to biotic stress involves a few steps: after the initial signal input from the pathogen which is recognized by the related receptors (putative R genes), transcription of the cascade of the plant defence mechanism is triggered, including oxidative stress changes. Inside the cell, signals are transmitted to lead to the production of defence molecules (PR-proteins, heat shock proteins and secondary metabolites). Genes with experimental indication of involvement in the biotic stress are gathered on the main panel (coloured with dark grey), while genes and pathways that are putatively involved in biotic stress pathway are shown on the left and right sides (coloured in light grey). *a*) Potato samples 30 minutes after inoculation with potato virus *Y*. *b*) Tobacco samples 24 hours after inoculation with *M. secta*. In both cases, the signal after infection is expressed as a ratio relative to the signal in unifected controls, converted to a *log*_2 _scale, and displayed. The scale is shown in the figures.

In order to enable easier data visualization and interpretation of gene expression in potato – virus and other biotic interactions, the genes which had been classified as potentially being involved in biotic stress (BIN 20.1) were further subdivided into respiratory burst, receptors, signalling, kinases, regulation of transcription, heat shock proteins, pathogenesis-related (PR) proteins, secondary metabolism and miscellaneous functions. A sub-subBIN comprising proteinase inhibitors was added to a subBIN of PR proteins. New subBINs reflect the pathogen's signal transduction pathway and the plant's response to infection (Figure 1). Since some genes that are involved in response to stress, e.g. proteinase inhibitors, are involved in constitutive processes as well [15], the classification was based on the level of involvement of the gene (e.g. recognition, signalling etc.) rather than on its molecular function. 509 unigenes were mapped into BIN 20.1. Of those, slightly over 50% belong to receptors subBIN, consisting of several putative R (resistance) genes and 30% to subBINs consisting of PR proteins (Table 2).

**Table 2:** New organization for BIN 20.1. New subBINs for BIN 20.1 biotic stress and the percentage of genes, mapped to respective subBIN. Altogether the number of newly assigned clones to BIN 20.1. was 509, around half of those present on the 10 k potato microarray. The percentage of the clones from a particular subsubBIN compared to all the clones from subBIN 20.1 is shown.

**BIN code**	**BIN name**	**%**
20.1.01	stress.biotic.respiratory burst	1.4
20.1.02	stress.biotic.receptors	51.3
20.1.03	stress.biotic.signaling	7.3
20.1.04	stress.biotic.kinases	1.2
20.1.05	stress.biotic.regulation of transcription	2.0
20.1.06	stress.biotic.heat shock proteins	0.8
20.1.07	stress.biotic.PR-proteins	15.5
20.1.07.06	stress.biotic.PR-proteins.proteinase inhibitors	15.1
20.1.08	stress.biotic.secondary metabolites	1.2
20.1.09	stress.biotic.misc	4.3

	Σ stress.biotic	100

Proteinase inhibitors play an important role in plant defence mechanisms [15] against pathogens and pests. These were also mapped together and can now be found in BIN 29.5.15 (protein.degradation.inhibitors) which has 49 entries that were originally distributed between five proteinase BINs: cysteine, serine, aspartate, metalloprotease and ubiquitin. A large proportion of this BIN is also annotated to BIN 20.1.7.6, what was considered appropriate due to their involvement in "common" physiology as well as biotic stress responses.

This improvement of the structure of BIN 20.1 (biotic stress) provides a more detailed biotic stress scheme (Figure 1), allowing various stages of a plant's response to biotic stress can be investigated. Previously, biotic stress subBINs were not classified, which made it more difficult for the experimenter to differentiate between different stages of biotic stress response. Along with subBINs of the BIN 20.1, BINs containing genes that are putatively involved in plant response to pathogen or pest attack, were added to the scheme. These include genes involved in hormone signalling, signalling, redox state, cell wall, protein degradation [16], heat shock proteins [17] (BINs 17, 30, 21, 10, 29.5, 20.2.1 respectively), and a family of selected transcription factors [18]. Bin 20.2, containing genes involved in abiotic stress is also shown on the scheme, since it may contain general stress related genes.

### Mapping unassigned clones on the microarray

BIN 35 includes unknown clones having either

• no decision made about their ontology yet, despite having some annotation (subBIN 35.1),

• and having a low similarity to characterized Arabidopsis genes (subBIN 35.2).

From the 1582 clones assigned to BIN 35.1, that are represented on the potato 10 k microarray, 52 clones were assigned to corresponding BINs in the case where the potato annotation (together with high homology and coverage) was more specific than that for Arabidopsis. Of these 52 clones, eight were annotated to more than one subBIN. The majority of newly assigned clones belonged to BIN 20, which is not surprising since, in the current work, the emphasis was put on this group of genes.

The clones with E values > 10^-10 ^after BLASTing against the Arabidopsis protein set are candidates for unique potato genes. Initially they were all mapped to BIN 35.2, and we focused on classifying them, especially those present on the potato 10 k array. The clones were mapped to the appropriate BINs using TIGR annotation of the corresponding TC and NCBI BLASTx search results (nr database), together with literature survey.

From 4,687 clones that were originally mapped to BIN 35.2 and are present on the potato 10 k microarray,

• 2,972 had low similarity to Arabidopsis sequences – *group I*

• 1705 were similar to Arabidopsis sequences coding for proteins annotated only as "expressed_proteins", and that could not be mapped into other existing bins – *group II*

97 clones from subBIN 35.2 were mapped to more appropriate BINs. The majority of the newly mapped clones were potato or other Solanaceae-specific (Table 3). Taking into account our special focus on genes involved in the biotic stress, the majority of the newly annotated clones (35) were mapped to BIN 20.1 and its subBINs. Examples of Solanaceae specific transcripts include genes for metallocarboxypeptidase inhibitor, a potato PR-10a protein [19], several potato cysteine proteinase inhibitors [15] and potato polyphenol oxidase.

**Table 3:** New mappings for BIN 35.2. Similarity to Solanaceae and non-Solanaceae species for the 97 clones, initially from BIN 35.2 that were annotated to more appropriate BINs after additional BLASTing in nr database and literature survey. Group I denotes clones that had low similarity to Arabidopsis sequences while group II stands for sequences coding for proteins with significant similarity but with unknown function.

**specific for**	**group I**	**group II**	Σ
potato	43	5	48
tomato	17	4	21
other Solanaceae	9	0	9
*Arabidopsis*	6	1	7
other	7	5	12

Σ	82	15	97

### BIN representation on the potato microarray

Since TIGR potato 10 k cDNA microarrays are used by several researchers working on potato and other Solanaceae species, we investigated how various BINs and subBINs are represented on the potato microarray.

Globally, around 36% (15,817 out of 43,408) of potato sequences from the StGI database are present on the potato microarray. Twelve BINs out of 33 (see Table 1 in bold) are completely covered (BIN and all the subBINs) on the microarray. In other words, at least one clone from every BIN and its corresponding subBINs mentioned above are present on the microarray. There were only minor discrepancies such as (*i*) very small subBINs with few entries that had no 10 k microarray clone representative (e.g. 26.09 misc.glutathione S transferases with 1 entry), or (*ii*) half of a sub-subBIN is sometimes being missing on the microarray (e.g. subBIN 17.3.1.01 hormone metabolism.brassinosteroid.synthesis-degradation.reductase). This kind of discrepancy, where some subBINs are completely missing on the microarray while others are fully present, is usually found in sub-subBINs, representing single enzymatic functions, and not at the higher hierarchical BIN splits. Consequently, we can say that, in general, BIN representation is well covered on the TIGR microarray, enabling investigation of various physiological issues. Some improvements in this aspect could be incorporated in the next versions of TIGR potato microarrays.

### Experimental data

To present the functionality of potato mapping for MapMan, we explored two experiments, by which the complexity of the pathogen vs. plant interaction was assessed. Since plant responses to pathogen attack are still far from being completely understood [20], these experiments provide a useful insight into their reaction to pathogen infection.

A simple comparative design experiment was conducted in which, in the first experiment, a potato cultivar resistant to potato virus Y^*NTN *^(*PVY*^*NTN*^) was tested. To show the versatility of the biotic stress pathway, a second, previously published [21] experiment was reexamined, in which coyote tobacco (*Nicotiana attenuata*) plants were tested for their reaction to a herbivore insect, *Manduca secta*. The aim of both experiments was to investigate on plant's involvement in biotic stress response on the transcriptome level. In both experiments, plants were divided in two groups; being virus-inoculated or insect-treated and the other half being mock-inoculated or non-treated in the case of potato or tobacco experiment, respectively. Potato and tobacco plants were harvested 30 minutes and 24 hours post infection, respectively. While the potato experiment was performed in-house, the raw data for the tobacco experiment was downloaded from the SGED database, in which currently around 50 Solanaceae studies are deposited [22]. Three biological replicates for a virus and a mock infected sample were analysed using the TIGR potato 10 k microarray. Data were analyzed using R statistical software with the limma package [23]. The output of the analysis, a list of possible differentially expressed genes (*p *< 0.05), with their respective *M *values, was visualized using MapMan (Figure 1a and 1b). Instead of having to analyze each gene separately, the difference in expression can be now visualized for a whole BIN. Biological hypotheses can thus be confirmed or rejected, since a larger picture, that includes all the differentially expressed genes, is seen.

For the potato *PVY*^*NTN *^experiment, 575 clones were differentially expressed, of which 16 belong to the biotic stress response pathway (Figure 1a). It is easily seen that the clones belonging to the beginning (putative R genes) and the end (PR-proteins) of the pathway are mostly downregulated after viral infection. Moreover several other genes putatively involved in biotic stress (such as heat shock proteins and transcription factors), together with genes involved in abiotic stress, have mostly been downregulated. This intriguing result becomes immedeatly obvious after inspecting the provided figure or by looking at changed categories. These subBINs had a low p-value for Wilcoxon rank sum test which confirmes that the average response of a BIN is different from the response of all the other BINs [5]. It seems that 30 minutes post infection is too early for a plant to establish real defence response. At this time genes that are involved in other processes are upregulated (data not shown). This result is in concordance with previously published studies involving cucumber mosaic virus infection to *Arabidopsis thaliana *where at early stages of response, most of the significantly expressed genes were downregulated [24]. Additional experiments should be performed in order to confirm this hypothesis.

A list of 875 differentially expressed clones was obtained after *M. secta *attack on *N. attenuata*. Their involvement in biotic stress pathway is presented on Figure 1b. Here, in contrast to the potato experiment, genes that are represented on our biotic stress pathway (10 clones) are almost all upregulated. Moreover, several other genes that are deeply involved in plant-pest interaction, namely jasmonic acid pathway and genes involved in secondary metabolites synthesis were upregulated as discussed in [21]. However, whereas previously candidate genes had to be examined separately and functional classes had to be assigned based on the annotation, now this can be done with a few mouse-clicks by simple pasting lists [10,25]. As we see a quantitatively similar response as previously described, the adaptation of MapMan will help in classifying genes from potato and thus speed up array analysis significantly especially in the area of pathogen interaction. A more thorough examination of the results could lead to formation of interesting biological hypotheses on the mechanism of resistance/reaction to virus/insect infection. The observed difference in the two responses might be attributed to different experimental systems: different plants, stressors and most importantly, time after treatment. It seems that in early response to pathogen, plant defence have not yet been activated.

## Conclusion

Tools that integrate microarray data with biological processes in which the genes are involved have been developed mostly for human and microorganism microarrays. Consequently, they do not include processes and metabolic pathways specific for plants (e.g. photosynthesis) and they can-not be used to visualize data obtained by plant microarrays. Additionally, plants are remarkably diverse and therefore additional pathways may need to be implemented for each species. Tools that are specific for plant metabolism have been developed [2-4] but many of them have been Arabidopsis-specific. MapMan is different as it is more flexible, enabling any organism to be mapped to the existing MapMan ontologies. It has already been adapted for use with tomato [7] and Medicago [26]. With the implementation of potato mappings to MapMan, visualization and interpretation of more complex biological data from experiments performed on potato will be easier. As current trends in molecular biology are focused on connecting results from different levels of '-omics' data analysis (e.g. transcriptomic with metabolomic data analysis), this will be possible with potato experiments too, since MapMan enables the visualization of trancriptomic and metabolomic data simultaneously [5,6]. Conversly, it will be possible to use our improved dissection of biotic stress in BIN 20.1 and the schematic representation of the plant's response to infection for MapMan mappings in other species (Arabidopsis, tomato, Medicago). Thus the results of complex analysis can be interpreted on an additional level, and biotic stress responses in different experimental systems can be compared. This can increase the understanding of plant response to pathogen or pest attack.

## Methods

### BLAST

The sequences and the annotations of the potato gene index (*Solanum tuberosum *gene index, StGI) version 10 were downloaded from the StGI database (38,239 unique potato unigene identfiers). The unigenes sequences were then BLASTed (BLASTx, version 2.2.14) against Arabidopsis proteins release TAIR 6 (file TAIR6_pep_20051108, available from [27]) under default settings. In this way, every potato clone was assigned up to ten best matching Arabidopsis genes. The complete mapping file had 43,408 entries.

### Annotations

Following BLASTing, the file was modified in order to contain only the potato unigene name and its annotation from StGI, the protein domain description from StGI, if any, the best matching Arabidopsis gene names and the corresponding E value. The information on the presence of the clone on the TIGR potato 10 k microarray was included, where an extra column to the final output data was added, showing the potato clone name if present on the microarray. The BIN assignment from Arabidopsis was also included in order to match the original BIN assignment for every gene name.

### Manual annotation evaluation

Every potato clone annotation which was derived from its corresponding tentative contig, was checked manually in order to compare it with the description of the matching Arabidopsis entry. If it differed from the Arabidopsis description, the E value was checked. If the E value was reasonably low (threshold was chosen at 10^-15^), the BIN assignment was left as for Arabidopsis. When E values were higher than the chosen threshold (between 10^-10 ^and 10^-15^), clones were assigned manually to corresponding BINs, on the basis of sequence (NCBI BLASTx, nr database), protein domain information (PFAM [28], SMART [29]) and TIGR annotation. An additional literature survey was performed when needed. Where appropriate, BIN assignments were changed for original Arabidopsis mappings as well.

### Array analysis

A simple comparisons design analysis was applied (virus infected/insect treated versus mock inoculated/untreated samples). All calculations were done in limma software package for R with the limma package [23,30]. Two normalizations were performed, loess [31] and vsn [32]. The intersection of genes, resulting as differentially expressed both by applying loess and vsn normalization were used for further analysis.

### Adaptation of the MapMan ontology to biotic stress

BIN 20.1 (biotic stress) has been further divided into subBINs based on previous knowledge about plant's response to pathogen infection and pest attack.

New subBINs for proteinase inhibitors have been added to BIN 29, protein degradation.

Special attention was drawn to BINs 35.1 not assigned.no ontology and 35.2 not assigned.unknown. The latter consisted of clones with E values > 10^-10 ^after BLASTing. Clones from this BIN that are present on the TIGR potato 10 k microarray were checked manually and, where appropriate, were assigned to corresponding BINs as described above.

### Pictorial representation of stress responses in MapMan

A new biotic stress pathway showing also putative involvement in stress response was constructed using our in-house expert knowledge about the involved stress processes [33]. BINs were assigned to each part of the pathway after importing the image to MapMan. The procedure is described in [5]. In short, a mouse click on the selected location on the pathway opens a pop-up dialog box where the BIN and subBIN codes are entered. This is repeated for all BINs and subBINs that are to be represented on the display. The resulting assignments are stored as an .xml file which is linked to the map image.

## Competing interests

The author(s) declare that they have no competing interests.

## Authors' contributions

AR participated in performing BLAST and initial annotations, did most of the manual annotations and corrections, participated in designing biotic stress pathway, did the statistical analysis and wrote the paper. BU did BLAST and initial annotations, confirmed the proposed corrections and put expert knowledge into mapping the clone names. ŠB performed the biological experiment, participated in manual annotation, especially focusing on BINs 20.1, 29.5.15 and 35.2 and organized the biotic stress pathway. MS put expert biological knowledge into the evaluation of the results. KG originated the study, put expert biological knowledge into certain mappings and designed the biotic stress pathway. All authors edited, read and approved the final manuscript.
